# High Serum Lipopolysaccharide-Binding Protein Level in Chronic Hepatitis C Viral Infection Is Reduced by Anti-Viral Treatments

**DOI:** 10.1371/journal.pone.0170028

**Published:** 2017-01-20

**Authors:** Hsiao-Ching Nien, Shih-Jer Hsu, Tung-Hung Su, Po-Jen Yang, Jin-Chuan Sheu, Jin-Town Wang, Lu-Ping Chow, Chi-Ling Chen, Jia-Horng Kao, Wei-Shiung Yang

**Affiliations:** 1 Graduate Institute of Clinical Medicine, College of Medicine, National Taiwan University, Taipei, Taiwan; 2 Liver Disease Prevention and Treatment Research Foundation, Taipei, Taiwan; 3 Department of Family Medicine, National Taiwan University Hospital, Taipei, Taiwan; 4 Department of Internal Medicine, National Taiwan University Hospital Yun-Lin Branch, Yun-Lin, Taiwan; 5 Department of Internal Medicine, National Taiwan University Hospital, Taipei, Taiwan; 6 Department of Surgery, National Taiwan University Hospital, Taipei, Taiwan; 7 Department of Microbiology, College of Medicine, National Taiwan University, Taipei, Taiwan; 8 Graduate Institute of Biochemistry and Molecular Biology, College of Medicine, National Taiwan University, Taipei, Taiwan; 9 Department of Medical Genetics, National Taiwan University Hospital, Taipei, Taiwan; 10 Graduate Institute of Epidemiology and Preventive Medicine, College of Public Health, National Taiwan University, Taipei, Taiwan; 11 Hepatitis Research Center, National Taiwan University Hospital, Taipei, Taiwan; Chiba University, Graduate School of Medicine, JAPAN

## Abstract

**Background:**

Lipopolysaccharide-binding protein (LBP) has been reported to associate with metabolic diseases, such as obesity, diabetes, and non-alcoholic fatty liver disease. Since chronic hepatitis C virus (HCV) infection is associated with metabolic derangements, the relationship between LBP and HCV deserves additional studies. This study aimed to determine the serum LBP level in subjects with or without HCV infection and investigate the change of its level after anti-viral treatments with or without interferon.

**Methods and Findings:**

We recruited 120 non-HCV subjects, 42 and 17 HCV-infected subjects respectively treated with peginterferon α-2a/ribavirin and direct-acting antiviral drugs. Basic information, clinical data, serum LBP level and abdominal ultrasonography were collected. All the subjects provided written informed consent before being enrolled approved by the Research Ethics Committee of the National Taiwan University Hospital. Serum LBP level was significantly higher in HCV-infected subjects than non-HCV subjects (31.0 ± 8.8 versus 20.0 ± 6.4 μg/mL; *p*-value < 0.001). After multivariate analyses, LBP at baseline was independently associated with body mass index, hemoglobin A1c, alanine aminotransferase (ALT) and HCV infection. Moreover, the baseline LBP was only significantly positively associated with ALT and inversely with fatty liver in HCV-infected subjects. The LBP level significantly decreased at sustained virologic response (27.4 ± 6.6 versus 34.6 ± 7.3 μg/mL, *p*-value < 0.001; 15.9 ± 4.4 versus 22.2 ± 5.7 μg/mL, *p*-value = 0.001), regardless of interferon-based or -free therapy.

**Conclusions:**

LBP, an endotoxemia associated protein might be used as an inflammatory biomarker of both infectious and non-infectious origins in HCV-infected subjects.

## Introduction

Lipopolysaccharide-binding protein (LBP) is a 50-kD polypeptide abundantly synthesized and secreted by the liver [1]. LBP binds the lipid A portion of lipopolysaccharide (LPS) and induces toll-like receptor-4 (TLR-4)/ myeloid differentiation protein-2 (MD-2)/ cluster of differentiation 14 (CD14) signal pathways in inflammatory reactions [2, 3]. Therefore, it plays an important role in innate immunity [2, 4, 5]. Measurement of blood LPS is difficult because of its short half-life and inconsistency with current available method [6]. In contrast, LBP is relatively stable in blood with a half-life of 12–24 hours and can be easily measured with immunoassay. Therefore, LBP can be used as a biomarker in place of LPS to represent endotoxemia of chronic low-grade inflammation [4, 6, 7].

Recent studies reveal that non-alcoholic fatty liver disease (NAFLD), obesity and metabolic diseases are associated with increased low-grade endotoxemia of LPS [8–11]. In the liver, LPS and LBP complex activates myeloid differentiation primary-response protein 88 (MyD88)-dependent signal pathway [2, 12], leading to the activation of transcription factors nuclear factor (NF)-κB and activator protein (AP)-1 in activated hepatic stellate cells [12]. The MyD88-dependent pathway activates interferon regulatory factor 1 (IRF1) that translocates into the nucleus and cooperates with interferon regulatory factor 3 (IRF3) inducing type I interferon induction (IFN-α/β production) in the murine bone-marrow derived dendritic cell model [13]. Therefore, IFN-α/β are activated in response to LPS [14–16]. This signal pathway controls the expression of many pro-inflammatory cytokines and other immune related genes found in NAFLD or nonalcoholic steatohepatitis (NASH) [10].

Patients with severe obesity were reported to have higher serum LBP level which can be reduced after bariatric surgery [17]. LBP is also demonstrated to associate with high sensitivity C- reactive protein (hs-CRP), insulin resistance, dyslipidemia, and cytokeratin-18 fragment level [11, 17]. Patients with NAFLD were also shown to have higher LBP especially when with steatohepatitis as compared with simple steatosis [11, 18].

Chronic hepatitis C virus (HCV) infection has higher prevalence of hepatic steatosis, type 2 diabetes mellitus (T2DM) and insulin resistance [19]. However, how LBP is related to HCV infection is not clear at present. In studies of the subjects with human immunodeficiency virus (HIV) co-infected HCV, the level of LBP is higher at baseline compared with healthy subjects [20], and is also higher in HIV/HCV co-infected patients compared with HIV infected patients [21]. Besides, LBP level was slightly reduced at following sustained virologic response (SVR) after peginterferon α-2a/ribavirin therapy (interferon-based therapy, PR therapy) in antiretroviral therapy (ART)-treated HIV/HCV co-infected patients [20]. Furthermore, the change of LBP after interferon-based therapy or direct-acting antiviral drugs therapy (interferon-free therapy, DAA therapy) in HCV-infected patients is still not known.

In this study, we investigated serum LBP levels in HCV and non-HCV subjects and correlated their levels with anthropometric and metabolic parameters. In addition, whether interferon-based or -free therapy would alter the serum levels of LBP in chronic HCV-infected patients was also explored.

## Materials and Methods

### Subjects

This case-control retrospective study included 120 subjects without HCV or hepatitis B virus (HBV) infection as controls and 42 HCV-infected subjects who were treated with interferon-based therapy. Non-HCV control subjects were enrolled from outpatient clinics of National Taiwan University Hospital (NTUH) and Good Liver Clinic. This is a heterogeneous population with comorbidities, such as T2DM, hyperlipidemia, hypertension, metabolic syndrome, obesity, and fatty liver. The HCV-infected subjects were recruited from the gastroenterological clinics of NTUH. One group of 42 patients (Genotype 1, 2 or 6) received 24 or 48 weeks of peginterferon α-2a/ribavirin therapy with sustained viral response (SVR). We did not enroll any non-SVR patient in this study. In addition, 17 HCV-infected patients receiving DAA therapy were also enrolled. Seven and nine of this DAA group with genotype 1b were respectively treated with Sofosbuvir/Ledipasvir for 12 weeks and Daclatasvir/Asunaprevir for 24 weeks. One of the DAA group with genotype 2a was treated with Sofosbuvir/Ribavirin for 12 weeks. All subjects with acute systemic inflammatory diseases, such as sepsis, cellulitis, pneumonia or recent bone fracture were excluded. The study protocols were reviewed and approved following approved guidelines by the Research Ethics Committee of the National Taiwan University Hospital (Protocol ID No. 201407032RIFB, No. 200911025R and No. 201205058RIC). All the subjects provided written informed consent before being enrolled.

Basic information and clinical data, such as gender, date of birth, body weight, body height, body mass index (BMI, weight in kilograms divided by height in meters squared), and waist circumference (cm, at a level midway between the lowest rib and the iliac crest) were collected. Obesity was defined as BMI≧27 according to the definition proposed by Department of Health and Welfare in Taiwan [22].

### Blood tests and abdominal ultrasound

Blood samples were collected and assayed for aspartate aminotransferase (AST), alanine aminotransferase (ALT), α-fetoprotein (AFP), hepatitis B surface antigen (HBsAg) and HCV antibody (anti-HCV); total cholesterol (T-CHO), triglycerides (TG), HDL cholesterol (HDL-C) and LDL cholesterol (LDL-C); fasting plasma glucose, hemoglobin A1c (HbA1c); hs-CRP, leukocyte count (WBC) and platelets, were assessed with the standard clinical automatic analyzers (Toshiba Automated Biochemical Analyzer, Otawara-shi, Tochigi, Japan; Abbott architect i system (Non-Sterile), Kallang Place, Singapore; Trinity Biotech Ultra 2, Kansas, MO, USA; Sysmex Automated Hematology Analyzer XN series, Kakogawa, Hyogo, Japan). Serum LBP levels were measured by human LBP enzyme-linked immunosorbent assay kit (Biometec, Greifswald, Germany) according to the manufacturer’s instructions. HCV-RNA was measured for HCV-infected subjects by using COBAS Ampliprep/TaqMan and docking station afterwards (Roche, Schlieren, Switzerland).

Fatty liver and liver cirrhosis were identified with ultrasound (Toshiba SSA-320A or SSA-660A or Aplio 300, Otawara-shi, Tochigi-ken, Japan) by well-trained specialists. The diagnostic criterion for fatty liver was increased echogenicity of the liver parenchyma greater than the kidney cortex and spleen parenchyma due to intracellular lipid accumulation [23]. The severity of fatty liver was divided into four grades: normal, mild, moderate and severe. Mild fatty liver was defined as increased echogenicity of the liver when compared with renal cortex and severe fatty liver was found only the main portal vein walls could be visualized with absence of all smaller portal venule walls and/or gross discrepancy of the increased hepatic to renal cortical echogenicity. Then, moderate fatty liver was intermediate between these [23–25]. In studies, the sensitivity and specificity of ultrasound exam in detecting fatty liver had been found to be 60%-94% and 84%-95%, respectively [24, 26, 27]. Liver cirrhosis or fibrosis was defined with coarse echotexture, nodular surface and irregularly narrowed right hepatic vein [28].

### Statistical analysis

Statistical analyses were performed using IBM SPSS Statistics software v19.0. Categorical data were analyzed by the *chi*-squared test, while continuous data were analyzed by Student’s *t*-test or ANOVA test. The data at baseline and SVR (defined as an undetectable serum HCV-RNA level at week 24 after the end-of treatment in interferon-based therapy and at week 12 after the end-of treatment in interferon-free therapy) were analyzed by paired *t*-test. The relationship between variables was analyzed by simple correlation and multivariate linear regression analyses. A *p*-value < 0.05 was considered significantly.

## Results

The demographic and laboratory data of 162 subjects (120 non-HCV subjects and 42 HCV-infected subjects) are shown in Table 1. At baseline the values of BMI, waist circumference, T-CHO, LDL-C, WBC, platelets and the percentage of subjects with fatty liver were significantly higher in non-HCV subjects than those in HCV-infected patients (Table 1). In contrast, the baseline values of fasting plasma glucose, ALT, AFP and liver fibrosis or cirrhosis were lower in non-HCV subjects (Table 1). Importantly, serum LBP level was significantly higher in HCV-infected subjects than non-HCV subjects with or without obesity (34.6 ± 7.3 versus 20.0 ± 6.4 μg/mL; *p*-value < 0.001, Table 1). Non-HCV subjects with obesity had higher LBP than non-HCV subjects without obesity (21.2 ± 5.9 versus 18.7 ± 6.7 μg/mL; *p*-value = 0.003). Moreover, the HCV subjects with normal ALT still had higher LBP than the non-HCV subjects with abnormal ALT (32.2±10.3 versus 20.8±8.3 μg/mL; *p*-value = 0.004, S1 Table), suggesting higher LBP is associated with HCV infection.

**Table 1:** The characteristics of non-HCV subjects and HCV-infected subjects before and after peginterferon α-2a/ribavirin therapy.

	Non-HCV			HCV(Baseline)	HCV(SVR)	*p*-value*	*p*-value#
Numbers of subjects	All(N = 120)	Non-Obesity(N = 56)	Obesity(N = 64)	N = 42	N = 42	-	-
**Gender (M/F)**	37/83	14/42	23/41	24/18	24/18	0.002**	-
**Age (years)**	47.0±11.4	48.6±10.9	45.7±11.7	54.9±10.9	55.9±10.9	< 0.001	-
**BMI (kg/m**^**2**^**)**	28.3±4.9	24.5±2.2	31.6±4.2	25.4±4.3	25.1±4.0	0.001	0.184
**WC (cm)**	94.5±12.0	86.6±8.6	101.3±10.3	86.5±11.8	87.9±10.3	< 0.001	0.194
**Fasting glucose (mg/dL)**	97.7±15.1	93.8±11.1	101.2±17.3	105.3±18.7	103.7±19.7	0.009	0.535
**HbA1C (%)**	5.7±0.5	5.6±0.4	5.8±0.5	5.8±0.8	5.8±0.6	0.133	0.270
**Total cholesterol (mg/dL)**	195.8±33.4	199.8±32.7	192.2±33.8	170.6±25.4	186.7±32.7	< 0.001	< 0.001
**Triglycerides (mg/dL)**	137.0±86.4	122.2±68.4	150.0±98.2	119.9±78.4	136.9±77.1	0.261	0.025
**HDL cholesterol (mg/dL)**	49.5±11.2	54.2±11.2	45.4±9.5	46.8±14.3	47.6±11.2	0.216	0.660
**LDL cholesterol (mg/dL)**	121.5±34.4	121.7±34.5	121.4±34.6	99.5±23.9	116.3±29.7	< 0.001	< 0.001
**Leukocyte count**	6486±1783	6055±1464	6864±1956	5175±1423	5430±1618	< 0.001	0.167
**hs-CRP**	0.13±0.36	0.10±0.41	0.16±0.31	-	-	-	-
**Platelets (K/μL)**	284.3±64.0	274.8±57.0	292.6±68.8	186.2±60.1	182.1±55.2	< 0.001	0.457
**HCV RNA (log IU/mL)**	-	-	-	5.74±1.19	undetectable	-	< 0.001
**ALT (U/L)**	39.1±29.2	31.4±24.0	45.9±31.8	124.3±100.7	26.0±14.8	< 0.001	< 0.001
**AFP (ng/mL)**	3.1±1.7	3.5±2.0	2.7±1.2	10.4±16.8	3.6±2.4	0.007	0.012
**LBP (μg/mL)**	20.0±6.4	18.7±6.7	21.2±5.9	34.6±7.3	27.4±6.6	< 0.001	< 0.001
**Fatty liver (Y/N)**	106/14	43/13	63/1	25/17	24/18	< 0.001**	-
**Liver fibrosis & cirrhosis (Y/N)**	0/120	0/56	0/64	22/20	22/20	< 0.001**	-

Abbreviations: HCV, hepatitis C virus; BMI, body mass index; WC, waist circumference; HbA1C, hemoglobin A1c; HDL, high-density lipoprotein; LDL, low-density lipoprotein; ALT, alanine transaminase; AFP, alfa-fetoprotein; LBP, lipopolysaccharide binding protein; SVR, sustained virologic response; -: no data; Y/N: Yes/No.

* Comparison between non-HCV subjects and HCV-infected subjects at baseline. *p*-value is tested by *t* test

** *p*-value is tested by *x*^2^ test.

# Comparison between HCV-infected subjects at baseline and SVR. *p*-value is tested by paired *t* test.

In multivariate linear regression analyses of 120 non-HCV subjects and 42 HCV-infected subjects before interferon-based therapy shown in Table 2, serum LBP was significantly associated with BMI, HbA1c, ALT and HCV infection (Table 2). In non-HCV subjects, BMI and HbA1c were the significant factors associated with serum LBP concentration. Interestingly, serum LBP level in HCV-infected subjects was related only to ALT and fatty liver before and only to HbA1c and fatty liver after the interferon-based therapy (Table 2).

**Table 2:** Multivariate linear regression analyses with circulating LBP as dependent variable.

Numbers of subjects	ALL (N = 162)		Non-HCV (N = 120)		HCV(Baseline) (N = 42)		HCV(SVR) (N = 42)	
	β (SE)	*p*-value	β (SE)	*p*-value	β (SE)	*p*-value	β (SE)	*p*-value
**Age (years)**	0.008 (0.047)	0.867	0.027 (0.055)	0.628	-0.071 (0.106)	0.509	-0.100 (0.087)	0.259
**Gender (M/F)**	-0.833 (1.122)	0.459	0.208 (1.381)	0.881	-3.071 (2.474)	0.224	0.901 (1.943)	0.646
**BMI (kg/m**^**2**^**)**	**0.283 (0.114)**	**0.014**	**0.306 (0.131)**	**0.022**	0.099 (0.302)	0.744	0.387 (0.265)	0.153
**HbA1C (%)**	**2.598 (0.914)**	**0.005**	**2.992 (1.315)**	**0.025**	2.906 (1.501)	0.062	**4.039 (1.604)**	**0.017**
**LDL cholesterol (mg/dL)**	-0.008 (0.016)	0.594	-0.006 (0.017)	0.748	-0.005 (0.047)	0.918	0.049 (0.031)	0.119
**Leukocyte count**	0.001 (0.000)	0.057	0.001 (0.000)	0.091	0.001 (0.001)	0.264	0.001 (0.001)	0.454
**HCV RNA (log IU/mL)**	-	-	-	-	1.098(0.959)	0.261	-	-
**ALT (U/L)**	**0.022 (0.009)**	**0.017**	-0.001 (0.024)	0.980	**0.032 (0.012)**	**0.014**	0.045 (0.071)	0.526
**Fatty liver (Y/N)**	-2.447 (1.393)	0.081	-0.394 (1.916)	0.838	**-5.393 (2.256)**	**0.023**	**-4.672 (2.084)**	**0.032**
**HCV (Y/N)**	**12.990 (1.577)**	**<0.001**	-	-	-	-	-	-
**Intercept**	-4.388 (5.952)	0.462	-9.589 (7.845)	0.224	8.529 (12.006)	0.483	-7.691 (10.454)	0.467
**Adjusted R**^**2**^	0.552		0.112		0.142		0.308	

Abbreviations: SE: standard error; HCV, hepatitis C virus; BMI, body mass index; HbA1c, hemoglobin A1c; ALT, alanine transaminase; LBP, lipopolysaccharide binding protein; SVR, sustained virologic response; Y/N: Yes/No; -: no data. Statistically significant values are indicated in bold.

Furthermore, the change of serum LBP concentration in interferon-based therapy group was only significantly correlated with the change of HDL-C in simple correlation (*r* = 0.329, *p*-value = 0.033). The change of ALT, HCV-RNA, AFP, WBC, platelets, fasting plasma glucose, HbA1c, T-CHO, TG or LDL-C was not significantly correlated with the change of LBP.

Besides, the serum LBP level in HCV-infected subjects after receiving interferon-based therapy was significantly reduced to 27.4 ± 6.6 μg/mL (*p*-value < 0.001, Table 1). The change of LBP level was -7.2 ± 8.0 μg/mL. According to liver fibrosis and cirrhosis in HCV-infected subjects, S2 Table was modified from Table 1. In cirrhotic patients, LBP levels were slightly higher at baseline (35.4±7.0 versus 33.6±7.6 μg/mL, *p*-value = 0.432, S2 Table) and SVR (28.2±5.5 versus 26.4±7.7 μg/mL, *p*-value = 0.383, S2 Table). The serum LBP levels were significantly reduced from 33.6±7.6 to 26.4±7.7 μg/mL in non-cirrhosis group and from 35.4±7.0 to 28.2±5.5 μg/mL in cirrhosis group (*p*-value < 0.001 and 0.002, S2 Table). Similarly, HCV-RNA viral load, ALT, AFP and BMI were also significantly decreased, whereas T-CHO, TG and LDL-C were mildly elevated after interferon-based therapy (Table 1).

**Fig 1:**
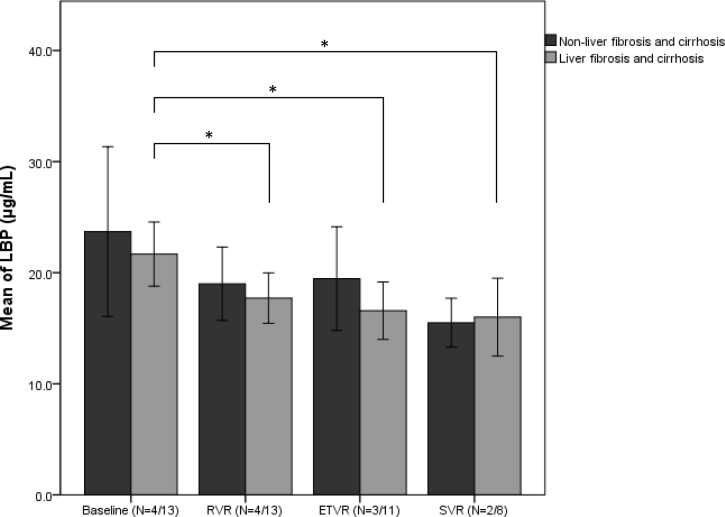
The serum levels of lipopolysaccharide-binding protein (LBP) at different stages in HCV-infected subjects treated with direct-acting antiviral drugs (DAA therapy). Abbreviations: Baseline: before DAA therapy; RVR: Rapid virologic response, defined as an undetectable serum HCV-RNA level at week 4 of treatment; ETVR: End-of treatment virologic response, defined as an undetectable serum HCV-RNA level at the end-of treatment; SVR: sustained virologic response, defined as an undetectable serum HCV-RNA level at week 12 after the end-of treatment. * *p*-value is < 0.05 tested by paired *t* test.

In the other 17 HCV-infected subjects treated with interferon-free drugs, the serum LBP levels were also significantly reduced from 22.2 ± 5.7 μg/mL to 15.9 ± 4.4μg/mL at SVR. The change of LBP level was -6.8 ± 4.6 μg/mL. As compared with the interferon-based therapy group, the change of LBP in the interferon-free therapy group had no significant difference (*p*-value = 0.872). When both HCV groups either with interferon-based or -free therapy were pooled together, the LBP level significantly declined from 32.3 ± 8.5 to 25.2 ± 7.7 μg/mL with therapies (*p*-value < 0.05). In the follow-up of the patients with interferon-free therapy, LBP levels gradually declined from the baseline to SVR with DAA therapy (22.2 ± 5.7 to 15.9 ± 4.4 μg/mL, *p*-value < 0.05). According to liver fibrosis and cirrhosis, LBP levels were both gradually declined especially in liver fibrosis and cirrhosis group (tested by ANOVA test, *p*-values = 0.023, Fig 1). However, LBP levels were slightly increased in non-liver fibrosis and cirrhosis group that might be the limited sample size.

## Discussion

In this study, we explored the relationship of LBP with metabolic parameters and HCV infection. We found that the serum LBP level was higher in HCV-infected subjects than that in non-HCV subjects. The LBP level was associated with HCV infection, ALT, BMI and HbA1c in multivariate linear regression analyses. In HCV-infected subjects, the LBP levels declined significantly after interferon-based or -free therapy.

The positive correlation with LBP and BMI had been clearly shown in several studies in non-HCV subjects [6, 17, 29, 30]. We also found independent association of LBP with HbA1c in non-HCV subjects and HCV-infected subjects after interferon-based therapy. Previously, the positive association between circulating LBP and HbA1c has been reported by others [31]. The association of these two metabolic factors with LBP is consistent with the notion of long postulated chronic low grade endotoxemia in metainflammation [17, 29–31]. Interestingly, the relationship between LBP and BMI or HbA1c disappeared in HCV-infected subjects before interferon-based therapy in this study. These results suggested that the rise of LBP serum level might be derived from at least two main sources. One was the HCV infection or liver inflammation and the other was the metabolic origin related to obesity and glycemia. The effect of HCV infection or liver inflammation appears more profound than the metabolic origins in term of LBP levels.

Jirillo et al. showed serum levels of sCD14 are higher in chronic HCV-infected subjects than those in normal subjects [32] and summarized the LPS concentrations of HCV-infected subjects who are the responders to PR therapy are significantly decreased while the LBP levels of 48% non-responders are still elevated [33, 34]. The interaction between LPS and HCV-associated pathogenesis might be multifactorial [35] including the impairment of T-cell-mediated antibacterial functions [36–38]; the leakage of endotoxins into the body due to increased intestinal permeability [39]; decreased hepatic detoxication of endotoxin because of HCV infection [39]. Therefore, the endotoxemia in HCV-infected subjects may be exogenous or endogenous [35]. In our study, we also observed the endotoxemia related marker, LBP, derived from two sources was higher in HCV-infected subjects.

Whether antiviral therapies for HCV infection can alter the serum levels of LBP was only investigated in HIV/HCV co-infected patients. High baseline level of LBP was associated with non-response to PR therapy and its level can be reduced by ART [21]. The other study also showed that LBP level at SVR was significantly decreased from baseline level after PR therapy in ART-treated HIV/HCV co-infected patients [20]. Our study showed high serum LBP level was reduced either after interferon-based or interferon-free therapy in HCV-infected subjects. However, we did not enroll non-responders in this study so that the changes of LBP level in responders could not be compared with those in non-responders. This limitation can only be supplemented by future studies.

The mechanism underlying the rise of LBP in HCV infection is not clear at present. Many organs are known to contribute to the synthesis of LBP, such as liver, gastro-intestinal tract, lung, kidney, and reproductive tract [4]. Previous studies showed that LBP was significantly correlated with inflammatory biomarker, hs-CRP, in obese subjects [17, 40]. In this study, LBP in HCV-infected subjects was only associated with ALT and fatty liver before and only with HbA1c and fatty liver after interferon-based therapy in multivariate linear regression analyses. Furthermore, the LBP level was reduced by anti-viral treatment, suggesting that the rise of LBP may indicate the inflammatory status in the liver.

We found the serum level of LBP was reduced with interferon-based therapy in HCV-infected subjects. However, since interferon-free therapy has become mainstream treatment for HCV worldwide [41], we therefore included a small group with DAA therapy. LBP was also reduced with interferon-free therapy. The magnitude of LBP change was similar between interferon-based and -free therapy groups. In addition, we also observed that LBP already significantly dropped with accompanying undetectable HCV-RNA viral load and diminished ALT after interferon-free therapy at 1 month and at the stage of RVR. These suggest that as ALT, LBP might serve as another important hepatic inflammatory biomarker in chronic HCV infection. Furthermore, the LBP levels of the DAA group before treatments were almost the same as those of non-HCV subjects with obesity. This might be caused by the very small sample size of the DAA group and that 41% of them were previously treated with PR therapy.

Several studies showed NAFLD patients had increased endotoxin levels in serum [42–44]. Moreover, the endotoxemia marker, LBP, was also associated with NAFLD in general population [11]. However, in our study, LBP was not associated with fatty liver, fibrosis or cirrhosis in all subjects after multivariate linear regression analyses. Fatty liver was not significantly associated with LBP in non-HCV subjects even with obesity. This might be caused by the small sample size of this study. Moreover, in HCV-infected subjects, the mean of LBP level was slightly lower in fatty liver group than that in non-fatty liver group (33.5±6.9 versus 36.1±7.7 μg/mL, *p*-value = 0.253). After adjusting multiple factors, the negative association between fatty liver and LBP was increased. When adding HbA1C as one of the adjusting factors, a significant *p*-value was found. The pathogenesis of fatty liver in HCV-infected subjects was multifactorial. The mechanisms of association between low LBP and fatty liver needs to be clarified and investigated in further studies with the larger sample size.

In conclusions, the serum level of LBP was significantly higher in HCV-infected subjects than in non-HCV subjects with or without obesity. Anti-viral treatments with or without interferon significantly reduced the LBP levels, suggesting a hepatic origin of LBP in HCV infection-related inflammation. In contrast, the association of LBP with BMI or HbA1c in non-HCV subjects suggests the other origin of LBP in metainflammation. All together, LBP may serve as an inflammatory biomarker for both infectious and non-infectious origins in HCV-infected subjects.

## Supporting Information

S1 STROBE Checklist STROBE Statement. (DOC)

S1 Table The levels of alanine aminotransferase (ALT) and lipopolysaccharide binding protein (LBP) in non-HCV subjects with abnormal ALT and HCV-infected subjects with normal ALT. (DOCX)

S2 Table The characteristics of HCV-infected subjects before and after peginterferon α-2a/ribavirin therapy. (DOCX)
