# Supplementary material for: High Serum Lipopolysaccharide-Binding Protein Level in Chronic Hepatitis C Viral Infection Is Reduced by Anti-Viral Treatments
Source: PLoS One. 2017 Jan 20;12(1):e0170028. doi: 10.1371/journal.pone.0170028 (PMC5249206; doi:10.1371/journal.pone.0170028)
Supplement: S1 Table — (DOCX) [file pone.0170028.s002.docx]

**S1 Table. The levels of alanine aminotransferase (ALT) and lipopolysaccharide binding protein (LBP) in non-HCV subjects with abnormal ALT and HCV-infected subjects with normal ALT**

|  | **non-HCV with abnormal ALT (≧40 U/L)** | **HCV with normal ALT (<40 U/L)** | ***p*-value*** |
| --- | --- | --- | --- |
| **Number** | 39 | 6 |  |
| **ALT (U/L)** | 72.7±29.1 | 31.7±6.0 | <0.001 |
| **LBP (μg/mL)** | 20.8±8.3 | 32.2±10.3 | 0.004 |

Abbreviations: HCV, hepatitis C virus; ALT, alanine transaminase; LBP, lipopolysaccharide binding protein

* p-value is tested by t test
