# Supplementary material for: High Serum Lipopolysaccharide-Binding Protein Level in Chronic Hepatitis C Viral Infection Is Reduced by Anti-Viral Treatments
Source: PLoS One. 2017 Jan 20;12(1):e0170028. doi: 10.1371/journal.pone.0170028 (PMC5249206; doi:10.1371/journal.pone.0170028)
Supplement: S2 Table — (DOCX) [file pone.0170028.s003.docx]

**S2 Table. The characteristics of HCV-infected subjects before and after peginterferon α-2a/ribavirin therapy**

|  | **HCV(Baseline)** | | | **HCV(SVR)** | | | ***p*-value*** | ***p*-value#** |
| --- | --- | --- | --- | --- | --- | --- | --- | --- |
| **Numbers of subjects** | **All N=42** | **Non- F & C N=20** | **F & C N=22** | **All N=42** | **Non- F & C N=20** | **F & C N=22** |  |  |
| **Gender (M/F)** | 24/18 | 13/7 | 11/11 | 24/18 | 13/7 | 11/11 | - | - |
| **Age (years)** | 54.9±10.9 | 50.2±11.4 | 59.1±8.7 | 55.9±10.9 | 51.3±11.3 | 60.2±8.7 | <0.001 | <0.001 |
| **BMI (kg/m^2^)** | 25.4±4.3 | 24.3±4.1 | 26.5±4.2 | 25.1±4.0 | 23.8±4.2 | 26.3±3.6 | 0.157 | 0.572 |
| **WC (cm)** | 86.5±11.8 | 83.7±13.4 | 89.1±9.6 | 87.9±10.3 | 85.1±10.6 | 90.5±9.6 | 0.379 | 0.357 |
| **Fasting glucose (mg/dL)** | 105.3±18.7 | 96.6±12.1 | 113.2±20.2 | 103.7±19.7 | 96.2±12.5 | 110.6±22.6 | 0.861 | 0.550 |
| **HbA1C (%)** | 5.8±0.8 | 5.6±0.3 | 6.1±0.9 | 5.8±0.6 | 5.5±0.4 | 6.0±0.7 | 0.163 | 0.454 |
| **Total cholesterol (mg/dL)** | 170.6±25.4 | 173.7±30.0 | 167.8±20.9 | 186.7±32.7 | 189.2±38.0 | 184.5±27.7 | 0.002 | 0.014 |
| **Triglycerides (mg/dL)** | 119.9±78.4 | 127.3±107.4 | 113.2±38.4 | 136.9±77.1 | 147.6±102.2 | 127.2±44.4 | 0.141 | 0.071 |
| **HDL cholesterol (mg/dL)** | 46.8±14.3 | 44.7±10.2 | 48.8±17.2 | 47.6±11.2 | 45.3±8.2 | 49.6±13.3 | 0.725 | 0.768 |
| **LDL cholesterol (mg/dL)** | 99.5±23.9 | 100.8±29.2 | 98.3±18.4 | 116.3±29.7 | 119.2±33.5 | 113.6±26.3 | 0.002 | 0.006 |
| **Leukocyte count** | 5175±1423 | 5266±1530 | 5094±1350 | 5430±1618 | 5495±1809 | 5371±1464 | 0.387 | 0.295 |
| **Platelets (K/μL)** | 186.2±60.1 | 204.7±63.2 | 169.4±53.3 | 182.1±55.2 | 207.2±60.0 | 159.3±40.0 | 0.658 | 0.284 |
| **HCV RNA (log IU/mL)** | 5.74±1.19 | 5.62±1.38 | 5.85±1.00 | undetectable | undetectable | undetectable | <0.001 | <0.001 |
| **ALT (U/L)** | 124.3±100.7 | 98.2±78.5 | 148.0±114.0 | 26.0±14.8 | 16.4±7.1 | 34.7±14.7 | <0.001 | <0.001 |
| **AFP (ng/mL)** | 10.4±16.8 | 6.4±8.0 | 14.0±21.6 | 3.6±2.4 | 2.8±10. | 4.4±3.1 | 0.049 | 0.049 |
| **LBP (μg/mL)** | 34.6±7.3 | 33.6±7.6 | 35.4±7.0 | 27.4±6.6 | 26.4±7.7 | 28.2±5.5 | <0.001 | 0.002 |
| **Fatty liver (Y/N)** | 25/17 | 9/11 | 16/6 | 24/18 | 7/13 | 17/5 | 0.687** | 1.000** |

Abbreviations: Non- F & C, non-liver fibrosis & cirrhosis subjects; F & C, liver fibrosis & cirrhosis subjects; HCV, hepatitis C virus; BMI, body mass index; WC, waist circumference; HbA1C, hemoglobin A1c; HDL, high-density lipoprotein; LDL, low-density lipoprotein; ALT, alanine transaminase; AFP, alfa-fetoprotein; LBP, lipopolysaccharide binding protein; SVR, sustained virologic response; -: no data; Y/N: Yes/No

* Comparison between the values at baseline and SVR in non-liver fibrosis & cirrhosis subjects. *p*-value is tested by paired *t* test.

# Comparison between the values at baseline and SVR in liver fibrosis & cirrhosis subjects. *p*-value is tested by paired *t* test.

** *p*-value is tested by McNemar test. In non-liver fibrosis & cirrhosis subjects, 2 subjects without fatty liver at baseline had fatty liver at SVR; 4 subjects with fatty liver at baseline didn’t have fatty liver at SVR; 14 subjects’ outcomes were the same. In liver fibrosis & cirrhosis subjects, 1 subject without fatty liver at baseline had fatty liver at SVR; no subject with fatty liver at baseline had different outcome at SVR; 21 subjects’ outcomes were the same.
